# Neighborhood Built Environment and Transport and Leisure Physical Activity: Findings Using Objective Exposure and Outcome Measures in New Zealand

**DOI:** 10.1289/ehp.1104584

**Published:** 2012-03-28

**Authors:** Karen Witten, Tony Blakely, Nasser Bagheri, Hannah Badland, Vivienne Ivory, Jamie Pearce, Suzanne Mavoa, Erica Hinckson, Grant Schofield

**Affiliations:** 1 Center for Social and Health Outcomes Research and Evaluation (SHORE) and Whariki Research Centre, School of Public Health, Massey University, Auckland, New Zealand; 2 Health Inequalities Research Programme, Department of Public Health, University of Otago, Wellington, New Zealand; 3 McCaughey VicHealth Centre for the Promotion of Mental Health and Community Wellbeing, Melbourne School of Population Health, University of Melbourne, Melbourne, Australia; 4 Centre for Physical Activity and Nutrition, National Institute of Public Health and Mental Health Research, Auckland University of Technology, Auckland, New Zealand; 5 Centre for Research on Environment, Society and Health, School of Geosciences, University of Edinburgh, Edinburgh, United Kingdom

**Keywords:** active travel, built environment, epidemiology, geographic information systems, neighborhood, physical activity, urban design, walkability

## Abstract

Background: Evidence of associations between neighborhood built environments and transport-related physical activity (PA) is accumulating, but few studies have investigated associations with leisure-time PA.

Objective: We investigated associations of five objectively measured characteristics of the neighborhood built environment—destination access, street connectivity, dwelling density, land-use mix and streetscape quality—with residents’ self-reported PA (transport, leisure, and walking) and accelerometer-derived measures of PA.

Methods: Using a multicity stratified cluster sampling design, we conducted a cross-sectional survey of 2,033 adults who lived in 48 New Zealand neighborhoods. Multilevel regression modeling, which was adjusted for individual-level (sociodemographic and neighborhood preference) and neighborhood-level (deprivation) confounders, was used to estimate associations of built environment with PA.

Results: We found that 1-SD increases in destination access, street connectivity, and dwelling density were associated with any versus no self-reported transport, leisure, or walking PA, with increased odds ranging from 21% [street connectivity with leisure PA, 95% confidence interval (CI): 0%, 47%] to 44% (destination accessibility with walking, 95% CI: 17%, 79%). Among participants who self-reported some PA, a 1-SD increase in street connectivity was associated with a 13% increase in leisure PA (95% CI: 0, 28%). SD increases in destination access, street connectivity, and dwelling density were each associated with 7% increases in accelerometer counts.

Conclusions: Associations of neighborhood destination access, street connectivity, and dwelling density with self-reported and objectively measured PA were moderately strong, indicating the potential to increase PA through changes in neighborhood characteristics.

The sharp rise in chronic disease prevalence worldwide has focused public health attention on routine daily practices associated with energy intake and expenditure—what we eat and how we move—and the environmental factors influencing these behaviors. A number of studies have examined how neighborhood environments support or undermine health-related practices. With respect to how we move, evidence of a relationship between neighborhood built environments, transport-mode use (i.e., car, foot, bicycle), and levels of physical activity (PA) among residents is accumulating (Frank et al. 2005; Transportation Research Board and Institute of Medicine of the National Academies 2005).

Evidence suggests that transport-related PA (e.g., walking to work or to the grocery store) and leisure-time PA, such as jogging or waling in a park, are influenced by different built environmental characteristics (Brownson et al. 2009; Owen et al. 2007; Saelens and Handy 2008; Sallis et al. 2009a). Residents walk more for transport if they live in neighborhoods with higher-density housing, easier access to a range of destinations including public transportation, well connected street networks, and a mix of land-use zones. Conversely, living in sprawling, car-dependent neighborhoods contributes to less walking for transport (Frank et al. 2004; Sallis et al. 2009b). Whether the neighborhood built environment influences leisure-time PA is less clear (Saelens and Handy 2008). In studies using a common protocol, attributes of a more walkable neighborhood (higher street connectivity, land-use mix and dwelling density) have been positively associated with leisure-time PA in the United States (Sallis et al. 2009b) and Belgium (Van Dyck et al. 2010) but not in Australia (Owen et al. 2007). However, leisure-time PA has been more commonly associated with neighborhood features such as pleasant aesthetics and proximity to green space and recreational facilities than walkable neighborhood features (Giles-Corti et al. 2005; Owen et al. 2004; Wendel-Vos et al. 2007).

Objective measures of the built environment, which are derived from geographic information systems (GIS) and PA (using accelerometery), increasingly are used in studies of urban form and PA. Their use is an advance on self-reported measures as they overcome any dependent measurement error between exposure and outcome that may bias the overall association. However, variability in the type and use of measures across studies continues, and few studies use both objective exposure and outcome measures (Brownson et al. 2009; Saelens and Glanz 2009; Sallis 2009; Story et al. 2009). A further limitation of many studies is the omission of residents’ preference for a more or less walkable neighborhood as a potential confounder in the relationship between neighborhood type and PA (Frank et al. 2007; Transportation Research Board and Institute of Medicine of the National Academies 2005)— people who do more PA may self-select to live in neighborhoods with built environments that support the behavior, again inducing a spurious association in research studies.

Our study strengthens the evidence of a relationship between urban form and PA by *a*) including objectively derived measures of the built environment: street connectivity, dwelling density, land-use mix, destination accessibility, and streetscape quality using GIS and field audit methods; *b*) reporting on both self-reported transport-related PA, leisure-time PA, and total walking and objectively measured (accelerometer) PA; *c*) controlling for neighborhood preference; and *d*) allowing comparisons of strengths of association for multiple neighborhood characteristics with multiple types of PA.

## Materials and Methods

The Understanding the Relationship between Activity and Neighbourhoods (URBAN) study is a cross-sectional observational study that collected PA data from residents of randomly selected households in 48 neighborhoods. The neighborhoods, located in four New Zealand cities—Christchurch and Wellington and Waitakere and North Shore in the Auckland metropolitan area—were stratified by high and low walkability. The 2,033 adult participants were 20–65 years of age at the time the data were collected between April 2008 and September 2010. URBAN is a component of the multicountry International Physical Activity and Environment Network (IPEN) collaboration and uses IPEN protocols for exposure and outcome measures (IPEN 2001). Ethical approval was granted by Auckland University of Technology and Massey University ethics committees. Informed consent was provided by all participants.

**Figure 1:**
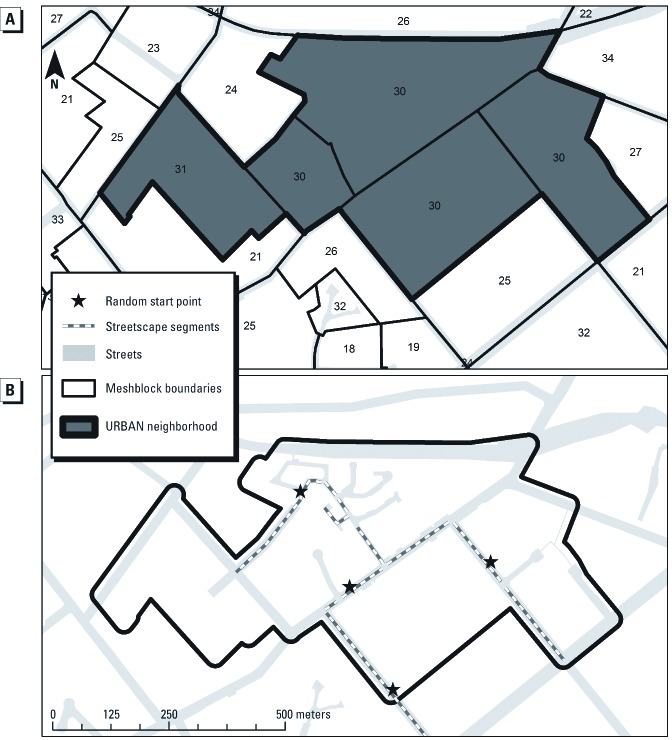
Illustration of an URBAN neighborhood comprising five adjacent meshblocks with high walkability scores (range, 4–40; *A*) and random start points for designated walks for participant recruitment and selected streetscape segments (*B*).

*Neighborhood selection.* A GIS-based walkability index (Leslie et al. 2007) was constructed for all meshblocks (small census areas of about 100 people) in each city. The index, which combined measures of dwelling density, land-use mix, street connectivity, and the retail floor area ratio, was generated using GIS software, ArcInfo 9.1 (ESRI, Redlands, CA, USA), according to IPEN research protocols (Badland et al. 2009; Frank et al. 2010; Leslie et al. 2007). Table 1 summarizes the data sources and GIS-based methods used to calculate the walkability index and exposure variables. Walkability index scores were used to select six high and six low walkability study neighborhoods per city. To maximize variability in the selected neighborhoods only meshblocks in the highest and lowest tertiles for walkability scores were eligible for selection. Each URBAN study neighborhood comprised five contiguous meshblocks, with consistently high or low walkability scores (Figure 1).

**Table 1:** Data sources and GIS method used in calculating walkability index and exposure measures.

Measure	Database	Data sourcea	Year	GIS-method
Street connectivity		Road center line		Territorial Local Authority		2007–2009		Intersection density is the number of intersections with ≥ 3 intersecting streets per square kilometer within a meshblockb
Dwelling density		New Zealand Census		Statistics New Zealand		2006		Number of dwellings per residential land area in a meshblock
		Land use and zoning		Territorial Local Authority		2007		
Land-use mix		Land use and zoning		Territorial Local Authority		2007		Entropy index based on presence or absence of five types of land use per meshblock
Retail floor area ratio		Building outline data		Territorial Local Authority		2007		Net retail area is the retail floor area divided by total retail parcel area within a meshblock
NDAI		Education facilities		Ministry of Education		2008		Accessibility is the either a presence or absence or an intensity measure of destinations (by type) accessible within walking distance (800 m along street network) of a meshblock centroid
		Public transit stops		Territorial Local Authority		2008		
		Green space and beaches		Ministry for Environment and Terra Link International		2005/2006		
		Social and cultural destinations, food outlets, financial services,		Internet, Territorial Local Authority, and GeoSmart		2008		
		Health facilities		Ministry of Health		2003		
aData held by Territorial Local Authorities were sourced from Waitakere City Council (Waitakere City, New Zealand), North Shore City Council (North Shore City, New Zealand), Wellington City Council (Wellington, New Zealand), and Christchurch City Council (Christchurch, New Zealand). New Zealand government agencies provided data on educational facilities (Ministry of Education, Wellington, New Zealand), health facilities (Ministry of Health, Wellington, New Zealand), national parks and reserves (Ministry for Environment, Wellington, New Zealand) and Census 2006 (Statistics New Zealand, Wellington, New Zealand). GeoSmart (Auckland, New Zealand) and TerraLink International (Wellington, New Zealand), private companies that supply geospatial information, provided data on sport facilities, churches, banks, and postal services. bMeshblock is the smallest unit for dissemination of New Zealand Census data with each unit representing approximately 100 people.								

*Participant recruitment.* Details regarding participant recruitment and data collection are described elsewhere (Badland et al. 2009). In brief, trained interviewers used a door-to-door recruitment strategy designed to achieve a representative sample of residents in each neighborhood. After enumerating all houses along designated walking paths, from random neighborhood start points, interviewers approached every *n*th house, where *n* varied from one to four according to neighborhood dwelling density. Interviewers made up to five return visits at different times of the day and on different days of the week to establish a household’s eligibility to participate and to recruit participants. One adult—the adult with the next birthday—was recruited per household. Participants were visited twice, 8 days apart, which enabled 7 days of PA data to be recorded. The study’s response rate was 44.8%.

*Built environment exposure measures.* Five built environment variables characterized neighborhood-level exposures—four GIS-derived variables and a systematic street audit. The GIS–derived measures (Table 1) were calculated at the meshblock level and the URBAN neighborhood score was derived as the mean value for the five constituent meshblocks in each neighborhood. Three of the GIS-derived variables were components of the walkability index used to select study neighborhoods–street connectivity, dwelling density, and land-use mix. The fourth GIS-derived measure was the Neighbourhood Destinations Accessibility Index (NDAI) (Witten et al. 2011)—a measure of walking access to 31 types of community service and amenity destinations to which proximity could plausibly encourage residents to walk more for leisure or transport. Educational, transport, recreation, social and cultural, food retail, financial, health, and other retail destinations were included (Witten et al. 2011).

A streetscape audit tool, the Systematic Pedestrian and Cycling Environment Scan (SPACES) (Pikora et al. 2002), was modified for use in New Zealand (Badland et al. 2010). The audit includes items that support walking and cycling such as physical infrastructure, street-level aesthetics and incivilities, and traffic-safety attributes. SPACES has demonstrated reliability for most items (Pikora et al. 2002). A trained field worker audited 12 street segments in each neighbourhood, providing a total of 576 audited street segments. Segments were selected sequentially from random neighbourhood start points (Figure 1). The values for the 12 audited segments were combined to provide a streetscape score for each neighbourhood.

*PA outcome measures.* Self-reported PA data were collected using the International Physical Activity Questionnaire–Long Form (IPAQ-LF) (Craig et al, 2003), and objective measures of PA were recorded using hip- mounted Actical accelerometers (Mini-Mitter, Sunriver, OR, USA). Using the IPAQ-LF, participants reported the number of hours and minutes they had engaged in specified PA domains in the 7 days before the trained interviewer’s second home visit. PA was delimited to light, moderate, and vigorous activity of at least 10-min duration. An outlier value for a self-reported PA was excluded for four participants. Accelerometer units were worn during waking hours on 7 consecutive days. The units were set up to record PA in 30 sec epochs.

Before analysis, we hypothesized three self-reported and two accelerometer-derived measures of PA to be responsive to neighborhood built environment features. The self-reported measures were IPAQ-LF minutes walking (overall total minutes walking for all purposes), IPAQ-LF minutes transport (overall total minutes of transport-related PA), and IPAQ-LF minutes leisure (overall total minutes leisure-time PA). Leisure-time PA encompasses activities performed for recreational purposes, whereas transport-related PA covers trips to and from utilitarian destinations.The reliability and validity of IPAQ-LF as a measure of PA engagement has been established in 12 countries, with correlations of around 0.8 for reliability and 0.30 for validity reported for the various PA domains (Craig et al. 2003).

The accelerometer-derived measures were the mean number of accelerometer counts recorded per hour while the accelerometer was worn during weekends and weekdays (weighted by hours of data recorded). Two rules were used to categorize data into wear- and nonwear blocks (De Bourdeaudhuij et al. 2003):

A period of > 59 min of consecutive zero counts signified a period of nonwear time, and the zeros were set to missing. This categorization was based on data streams and examination of counts.
A period of < 60 min during which the accelerometer was worn was also set to missing. This strategy was adopted in case participants only wore the accelerometer while exercizing, and eliminated counts from movement of an accelerometer when it was not being worn.

*Demographic, neighborhood preference, and neighborhood deprivation measures.* We collected information from participants on age, sex, ethnicity, marital status, household income, educational qualifications, occupation, household car access, and preferences for living in a more or less walkable neighborhood. Neighborhood preference was measured by presenting participants with illustrations and verbal descriptions of two types of neighborhoods—a lower-density suburban neighborhood located 10–15 min by car from common destinations and a higher-density urban neighborhood with most destinations accessible on foot or by public transportation within 10–15 min [see Levine and Frank (2007) for the neighborhood illustrations]. Participants were asked which of the two neighborhood types they would prefer to live in, and the strength of their preference, assuming housing cost, quality of schools and mix of people were similar in both neighborhoods. Responses were categorized using a five-point scale (strongly prefer walkable, moderately prefer walkable, neutral, moderately prefer less walkable, strongly prefer less walkable) (Levine et al. 2005). Using the New Zealand Deprivation Index 2006 (Salmond et al. 2007), study neighborhoods were categorized into quintiles by calculating the mean deprivation score of the five constituent meshblocks. All variables were identified *a priori* as potential confounders in the relationship between neighborhood built environment and PA.

*Statistical analysis.* We performed multilevel regression analyses using Stata (version 11.2; StataCorp LP, College Station, TX, USA) to allow for the hierarchical nature of the data (participants within neighborhoods within cities). In light of strong correlations between three built environment variables (destination accessibility, street connectivity, and dwelling density), each of the neighborhood exposures were modeled separately with each of the five outcome measures. The built environment exposures were also rescaled by dividing by their standard deviations (SDs) calculated across all neighborhoods. The major advantage of this rescaling is that the regression coefficients from models of different built environment exposures are more easily comparable, as they all refer to a 1-SD change. The data for the three specified IPAQ outcome domains—transport-related PA, leisure-time PA, and walking—had a bimodal distribution with 0 min of PA reported by many participants, while the remaining sample reported minutes of PA. Consequently, we used a two-step approach to analysis. First, for these three outcomes we undertook multilevel logistic regression models with *a*) any minutes versus *b*) no minutes of self-reported PA as outcome categories.

Second, for the accelerometer measures and for participants with non-zero IPAQ outcomes, we used linear multilevel regression analyses with the outcomes specified as the natural log of accelerometer counts and the natural log of IPAQ-LF minutes of PA (PA outcomes being positively skewed). Log transformation of all five outcome variables also greatly enhances comparison of model findings across outcome measures. That is, the regression coefficients when exponentiated are the ratio or relative increase in the outcome measure for each 1-SD change in the exposure variable. We report these results as ratios in tables (i.e., the regression coefficient exponentiated), but refer to the percentage changes in the text (e.g., a ratio of 1.13 is equivalent to a 13% increase in the outcome for a 1-SD change in exposure.)

All possible comparisons of exposure and outcome were assessed with four models: Model 1 was adjusted for sex, age, and ethnicity; model 2 was additionally adjusted for marital status, education, income, employment and car access (all individual or household-level covariates); model 3 was additionally adjusted for neighborhood-level deprivation; and model 4 was additionally adjusted for neighborhood preference. For estimates of the exposure–outcome associations for models 1, 2, and 3, see Supplemental Material, Tables 1 and 2 (http://dx.doi.org/10.1289/ehp.1104584). Model 4 estimates are reported here. Full model outputs, including coefficients for covariates and random error terms, are available on request. Covariates were modeled as categorical variables (Table 2).

**Table 2:** Participants by sociodemographics and New Zealand Deprivation Index 2006 categories.

Variable	n (%)	
Age (years)		
15–29	390	(22.0)
30–44	705	(39.0)
45–54	421	(23.0)
55–65	290	(16.0)
Ethnicity		
Maori	213	(12.0)
Non-Maori	1,593	(88.0)
Sex		
Male	773	(42.8)
Female	1,033	(57.2)
Qualification		
No high school qualification	469	(26.0)
High school qualification	206	(11.4)
Post-high school diploma or trade certificate	420	(23.2)
University degree	711	(39.4)
Marital status		
Never married	393	(22.0)
Married	1,159	(64.0)
Previously married	254	(14.0)
Household Income (NZ$)		
≤ 40,000	416	(23.0)
40,001–60,000	313	(17.3)
60,001–80,000	270	(15.0)
80,001–100,000	275	(15.2)
> 100,000	532	(29.5)
Employment		
Full time	1,080	(60.0)
Part time	468	(26.0)
Unpaid	258	(14.0)
Car access		
Unrestricted	1,485	(82.2)
Restricted	190	(10.5)
No car access	131	(7.3)
Preference		
Strongly prefer walkable	700	(34.9)
Moderately prefer walkable	333	(16.6)
Neutral	286	(14.3)
Moderately prefer less walkable	207	(10.3)
Strongly prefer less walkable	480	(23.9)
New Zealand Deprivation Index 2006		
Q1 (less deprived)	365	(20.2)
Q2	367	(20.3)
Q3	335	(18.5)
Q4	388	(21.5)
Q5 (most deprived)	351	(19.4)
Abbreviations: NZ$, New Zealand dollar; Q, quintile.		

## Results

The sociodemographic characteristics of the participants are described in Table 2. Women comprised 57.2% of the sample, and Maori made up 12%. Of the 86% of participants who were employed, 60% were working full time. Car access was high (82.2%), but neighborhood preference favored a more walkable environment. Summary statistics on the exposure and outcome variables are provided in Table 3. With regard to PA, a higher mean number of minutes of leisure-time PA (195 min) was reported than for transport-related PA (128 min). Accelerometer data show comparable levels of PA between weekdays and weekends.

**Table 3:** Exposure and outcome variables, URBAN study, 2008–2010.

Percentiles												
Variable	Meana ± SD		0	5	25	50	75	95	100
Neighborhood exposures																	
Dwelling density		5.87 ± 2.73			1		1.4		3.2		5.8		8		10		10
Street connectivity		5.42 ± 2.38			1		2.2		3.2		5.2		7.5		9		9.8
Mixed land use		5.68 ± 2.08			1.6		2.6		3.8		6		7.3		8.8		10
NDAI		11.74 ± 4.91			4.7		5.1		8.3		10.6		15.5		21.8		24.9
Streetscape		87.65 ± 11.31			68.6		72.9		79.4		84.9		94.8		111.4		121.9
PA outcomes																	
Transport time (min)		128 ± 260			0		0		0		60		150		420		5,040
Leisure time (min)		195 ± 268			0		0		0		120		275		670		3,360
Walk time (min)		438 ± 639			0		0		60		190		480		1,890		5,820
Weekday (acc)b		9,187 ± 4,992			290		3,257		5,752		8,294		11,561		18,024		45,287
Weekend (acc)		8,981 ± 5,687			44		2,566		5,109		7,789		11,405		19,592		45,356
acc, accelerometer. aNeighborhood exposures were calculated across study neighborhoods. bCounts per hour during the time the participant wore the accelerometer (weighted by hours of data recorded).																	

We observed reasonably high correlations between the neighborhood-level measures (Table 4), most notably between street connectivity, destination accessibility, and dwelling density. Neighborhood-level deprivation was moderately correlated with these three variables as well; for example, more deprived neighborhoods tended to have higher dwelling density, density of neighborhood destinations (i.e., NDAI), and street connectivity.

**Table 4:** Correlations between neighborhood-level characteristics (*n* = 48 neighborhoods).

Variable	Dwelling density	Street connectivity	Mixed land use	NDAI	Streetscape	NZDep06
Dwelling density	1.00					
Street connectivity	0.89*	1.00				
Mixed land use	–0.02	0.096*	1.00			
NDAI	0.71*	0.75*	0.097*	1.00		
Streetscape	0.18*	0.25*	0.34*	0.38*	1.00	
NZDep06	0.37*	0.42*	–0.004*	0.39*	0.018	1.00
NZDep06, New Zealand Deprivation Index 2006. *p < 0.01.						

Table 5 indicates a moderate correlation between self-reported transport and walking PA values and weekday and weekend accelerometer measures. Weak negative correlations were also evident between neighborhood deprivation and leisure-related PA and weekend accelerometer measures.

**Table 5:** Correlations between the PA outcome measures and neighborhood-level deprivation (*n* = 48 neighborhood).

Variable	Transport time	Leisure time	Walking time	Weekday acc	Weekend acc	NZDep06
Transport time	1.00					
Leisure time	0.19*	1.00				
Walking time	0.47*	0.35*	1.00			
Weekday acc	0.19*	0.28*	0.27*	1.00		
Weekend acc	0.15*	0.28*	0.18*	0.57*	1.00	
NZDep06	0.05	–0.14*	0.03	–0.009	–0.12*	1.00
Abbreviations: acc, accelerometer; NZDep06, New Zealand Deprivation Index 2006. *p < 0.01.						

Table 6 shows the odds ratios (ORs)for any versus zero self-reported minutes of transport-related PA, leisure-time PA, and total walking. Focusing on the fully adjusted model, 1-SD increases in destination accessibility and street connectivity were associated with any (versus no) self-reported transport-related PA, leisure-time PA, or walking, with increased odds ranging from 21% [for leisure-time PA with a 1-SD increase in street connectivity, 95% confidence interval (CI): 0%, 47%) to 44% (for total walking with a 1-SD increase in destination accessibility, 95% CI: 17%, 79%). Associations of the outcomes with streetscape, dwelling density, and mixed land use were weaker with the exception of streetscape with leisure-time PA where a 1-SD increase in streetscape was associated with a 31% increase in leisure-time PA (95% CI: 12%, 53%). It is interesting to note that the strength of the associations, with the exception of streetscape, tended to increase with adjustment for neighborhood deprivation—especially for leisure-time PA [see Supplemental Material, Table 1 (http://dx.doi.org/10.1289/ehp.1104584)]. That is, there was negative confounding by neighborhood deprivation, because higher neighborhood deprivation was associated with more walkable neighborhood built environments but also with less PA—especially leisure-time PA. Adjusting for neighborhood preference had little effect on odds ratios.

**Table 6:** ORs (95% CIs) in fully adjusted models for any self-reported transport, walking (all purposes), or leisure-related PA (vs. no self-reported PA), for a 1-SD change in each neighborhood exposure.

ORs for any self-reported PA versus none (95% CI)a				
Neighborhood exposure	Transport	Leisure	Walking
Adjusted for demographics, individual-level socioeconomic factors, neighborhood deprivation and neighborhood preferenceb		n = 1,779		n = 1,781		n = 1,778
Streetscape		1.13 (0.94, 1.35)		1.31 (1.12, 1.53)		1.11 (0.91, 1.36)
NDAI		1.39 (1.15, 1.69)		1.27 (1.06, 1.53)		1.44 (1.16, 1.79)
Street connectivity		1.42 (1.19, 1.69)		1.21 (1.00, 1.47)		1.32 (1.06, 1.63)
Dwelling density		1.36 (1.10, 1.67)		1.22 (0.99, 1.50)		1.27 (1.00, 1.59)
Mixed land use		1.03 (0.82, 1.29)		1.24 (1.02, 1.51)		1.02 (0.81, 1.30)
aFrom models of ln(IPAQ-minutes) on neighborhood exposures (transformed to have SD of 1.0) and covariates, whereby the exponential of the coefficient of the neighborhood exposure is the ratio change in any minutes of self-reported physical activity. bEstimates were generated using multilevel logistic regression models with the following covariates: age [15–29, 30–44 (reference group), 45–54, 55–65 years of age]; ethnicity [Maori, non-Maori (reference group)]; sex [male (reference group), female]; qualifications [no qualification, school, postschool, tertiary (reference group)]; marital status [never married, married (reference group), previously married]; household income [NZ$, < 40,000, 40,001–60,000, 60,001–80,000, 80,001–100,000, > 100,000 (reference group)]; employment [full-time work (reference group), part-time work, and not working]; car access [unrestricted (reference group), restricted, no car access]; neighborhood deprivation [New Zealand Deprivation Index 2006 quintile 1 (less deprived) (reference group) to quintile 5 (most deprived)]; and neighborhood preferences [strongly prefer walkable, moderately prefer walkable, neutral (reference group), moderately prefer less walkable, strongly prefer less walkable].						

Table 7 shows the ratio increases in self-reported minutes of transport-related PA, leisure-time PA, and total walking among participants who reported at least some PA and for accelerometer counts. In the fully adjusted models, a 1-SD increase in destination accessibility was associated with a 13% increase in total minutes walking (95% CI: 0%, 28%) and a 1-SD change in street connectivity was associated with a 14% increase in minutes of leisure PA (95% CI: 3%, 25%). In most cases associations for dwelling density, streetscape, and mixed land use with self-reported PA were weaker and mostly nonsignificant. We also found 1-SD increases in destination accessibility, street connectivity, and dwelling density were all associated with 7% increases in weekday accelerometer counts and with between 5% and 7% increases in weekend accelerometer counts (all *p* > 0.05). As with the logistic regression analyses, the associations for destination accessibility, street connectivity, and dwelling density tended to increase in magnitude, most notably for accelerometer counts, after adjusting for neighborhood deprivation [see Supplemental Material, Table 2 (http://dx.doi.org/10.1289/ehp.1104584)]. The shape of associations between neighborhood-level measures and PA outcomes were further investigated by adding a quadratic term in the full model, but we observed no improvement in fit compared with linear models.

**Table 7:** Predicted relative change (95% CI) in fully adjusted models of self-reported (among those with some self-reported activity) and accelerometer-measured PA, for a 1-SD change in neighborhood exposures.

Relative change in self-reported minutes of PA (95% CI)a					Relative change in accelerometer count		
Neighborhood exposure	Transport time	Leisure time	Walking time	Weekday	Weekend
Adjusted for demographics, individual-level socioeconomic factors, neighborhood deprivation and neighborhood preferenceb		n = 1,235		n = 1,315		n = 1,575		n = 1,619		n = 1,512
Streetscape		1.00 (0.92, 1.10)		1.09 (0.99, 1.19)		1.03 (0.92, 1.16)		1.03 (0.99, 1.07)		1.01 (0.97, 1.06)
NDAI		1.09 (0.98, 1.21)		1.12 (1.01, 1.23)		1.13 (1.00, 1.28)		1.07 (1.03, 1.11)		1.05 (1.00, 1.10)
Street connectivity		1.09 (0.98, 1.20)		1.14 (1.03, 1.25)		1.06 (0.93, 1.20)		1.07 (1.02, 1.11)		1.07 (1.02, 1.11)
Dwelling density		1.11 (0.99, 1.23)		1.09 (0.98, 1.22)		1.08 (0.94, 1.25)		1.07 (1.03, 1.12)		1.06 (1.02, 1.12)
Mixed land use		1.06 (0.95, 1.19)		1.10 (0.99, 1.22)		1.08 (0.94, 1.24)		1.03 (0.99, 1.08)		1.04 (0.99, 1.09)
aFrom models of ln(IPAQ-minutes) or ln(accelerometer count) on neighborhood exposures (transformed to have SD of 1.0) and covariates, whereby the exponential of the coefficient of the neighborhood exposure is the ratio change in accelerometer count. bEstimates were generated using multilevel linear regression models with the following covariates: age [15–29, 30–44 (reference group), 45–54, 55–65 years of age]; ethnicity [Maori, non-Maori (reference group)]; sex [male (reference group), female]; qualifications [no qualification, school, postschool, tertiary (reference group)]; marital status [never married, married (reference group), previously married]; household income [NZ$, < 40,000, 40,001–60,000, 60,001–80,000, 80,001–100,000, > 100,000 (reference group)]; employment [full-time work (reference group), part-time work, and not working]; car access [unrestricted (reference group), restricted, no car access]; neighborhood deprivation [New Zealand Deprivation Index 2006 quintile 1 (less deprived) (reference group) to quintile 5 (most deprived)]; and neighborhood preferences [strongly prefer walkable, moderately prefer walkable, neutral (reference group), moderately prefer less walkable, strongly prefer less walkable].										

## Discussion

Three objectively measured neighborhood characteristics—street connectivity, neighborhood destination access, and dwelling density—were positively associated with self-reported and accelerometer-derived measures of PA. A 7% increase in accelerometer-measured PA levels for each SD increase in the built environment measures was consistent across the three neighborhood measures for weekdays, with slightly lower values on weekend days for destination accessibility (5%) and dwelling density (6%). Land-use mix was associated with smaller (3–4%) but still significant increases in weekday and weekend accelerometer-derived PA activity. A comparison of findings for objective and self-reported (non-zero) measures of PA revealed a largely consistent pattern of positive relationships between neighborhood built environment exposures and PA domain outcomes. Of note, adjusting for neighborhood deprivation tended to increase the strength of association; in New Zealand poorer neighborhoods often have better overall access to destinations, and higher street connectivity and dwelling density than wealthier neighborhoods. The one exception to this negative confounding by neighborhood deprivation was transport-related PA, which is explicable in that individuals residing in poorer neighborhoods walk more for transport (Ministry of Transport 2009). More generally, the strength of associations between the objective exposure and outcome measures would have been biased to the null without adjusting for neighborhood deprivation.

The strengths of the present study were its large general population sample, as well as using neighborhoods from four cities, obtaining comparable numbers of participants who resided in higher and lower deprivation neighborhoods, using objective exposure and outcome measures, and adjusting for potential confounding by participants’ neighborhood preference. An additional strength was that our analysis enabled comparisons to be made between the magnitude of associations for specific neighborhood attributes with specific PA outcomes, both self-reported domains and objectively measured PA engagement. Including accelerometer data for all complete hours of wear time is a departure from usual protocols (e.g., Sallis et al. 2009b) that set minimum numbers of days and hours per day wear time criteria. We argue this departure strengthens the study because participants who are less compliant with the accelerometer protocol may differ systematically from those who are more compliant, and their exclusion may introduce bias to the study.

Despite the strengths noted, we also acknowledge that weaknesses remain. As a cross-sectional study, we did not have the ability to model temporality. In particular, distinguishing between the effect of neighborhood characteristics that influence PA and the effect of physically active people choosing to reside in particular neighborhoods is difficult. To address this concern, the ideal study would randomize individuals to different neighborhoods, which would provide a closer or less biased estimate of the causal association of built environment with PA. However, this approach is not feasible, raising the possibility of (residual) confounding by individuals’ preferences for, and self-selection into neighborhoods based on a desire to live in a more walkable neighborhood and, independent of neighborhood preference, on an individual’s level of PA (Frank et al. 2007). Our study adjusts at two levels for confounding by neighborhood preference. First, as the preference processes leading to this confounding are likely to be influenced by a range of sociodemographic characteristics (Frank et al. 2007), we included these factors as covariates in our analyses to adjust (at least partly) for such confounding. Second, and more directly, we adjusted for a measure of neighborhood preference and found associations were unchanged. For self-selection, neither Sallis et al. (2009b), who used objective measures of PA, nor Frank et al. (2007), who used self-reported measures, found self-selection explained positive associations with walkability. Contrary to these results, Owen et al. (2007) found evidence that self-selection attenuated the association between neighborhood walkability and PA for self-reported minutes of transport-related PA. In a meta-analysis of 52 multilevel built environment and travel studies in the urban planning and transport fields, Ewing (2009) found a small but nonsignificant effect of self-selection on walking for transport. Thus, we conclude that, for our study at least, residual confounding by neighborhood preference is unlikely to explain away our findings.

A second weakness of the URBAN study is the potential for selection bias because of an overall response rate of 44.8% [although this rate is higher than that found in other similar studies, for example, 26.0% (Frank et al. 2010) and 11.5% (Owen et al. 2007)]. For selection bias to arise, participation has to differ jointly or dependently by exposure and outcome, meaning that the exposure–outcome association differs between the nonparticipants and participants. Consider the 7% increase in accelerometer counts for a 1-SD increase in neighborhood built environment characteristics. If this association was truly null (i.e., 0%), the association among the nonparticipants would have to be [0.448 ÷ (1 – 0.448) × –7%] = –5.7% to account for the observed association (assuming no other sources of bias), such that walking would decrease in association with destination accessibility, street connectivity, and dwelling density among nonparticipants. Although we cannot rule it out, it seems unlikely that a reverse association of this magnitude would exist among the nonparticipants, and repeatedly so across multiple exposure–outcome associations.

For the exposure measures, the robustness of GIS-based indices is dependent on the quality of data common to jurisdictions involved in a study. Only relatively coarse common zoning data were available for the study’s four cities, which limited the sensitivity of the measure of land-use mix. However, this would most likely mean nondifferential misclassification bias of the neighborhood built environment, resulting in an underestimation of associations.

Although we investigated associations between the neighborhood built environment and residents’ PA, PA is likely to occur beyond, as well as within, the residential neighborhood. Future studies could improve internal validity by using global positioning to geographically locate where PA takes place. Further, models of activity–space exposure that incorporate the location and time of individuals’ daily movements (Chaix et al. 2009) could be usefully applied to determine how PA patterns vary for different population groups. Data on daily movements would help identify the most promising sites for built environment change to increase activity and to maximize population health gains.

The inclusion of neighborhoods in four cities is a noted strength of URBAN; however, the generally low density and limited variability of urban form in New Zealand reduces the generalizability of findings to cities with substantially different design attributes. The extent of variation in PA by the full range of variation in the built environment will only be established through the inclusion of cities with wide variation in built environment characteristics, an aim of the wider IPEN study. The separate effects of built environment variables on PA were investigated in the URBAN study. This contrasts with a common practice of combining measures of proximity (density and land-use mix) and connectivity (Frank et al. 2010; Leslie et al. 2007), into a walkability index for both neighborhood selection and modeling purposes. As URBAN’s findings illustrate, examining separate associations can identify variations as well as consistencies of effects—variations that may suggest alternative intervention pathways to promote transport-related PA or leisure-time PA in different urban settings. However, the correlation between these built environment attributes—both conceptually and statistically—render it difficult to identify which characteristic of the built environment is most important. Indeed, a regression model with all built environment attributes included as covariates will be limited by multicollinearity.

The URBAN study is among the first to use objective exposure and outcome measures to examine associations between the built environment and PA. As such, its findings add support to observations made in the United States and Belgium, which also were based on objective exposure and outcome measures, that higher street connectivity and dwelling density and more proximate access to amenities (Sallis et al. 2009b; Van Dyck et al. 2010) increased not only residents’ transport-related PA but also leisure-time and total PA outcomes.

*Implications for environmental health policy and practice.* Physical inactivity is a risk factor for many preventable diseases and chronic conditions. Despite the well-established health benefits of PA, more than half the adult population in New Zealand do not meet recommended levels of PA (Sport and Recreation New Zealand 2008) and similar figures apply in other industrialized countries. Increasing PA using individual behavior change interventions has met with limited success. In their meta-analysis of PA interventions, Conn et al. (2011) identified a mean difference of 14.7 min of PA per week between intervention and control groups. The estimated potential PA increases associated with a more walkable built environment make worthy comparisons. Estimates from our fully adjusted model suggest a mean population-level increase in minutes walking per week for a 1-SD built environment change of 57 min for destination accessibility, 26 min for street connectivity, and 35 min for dwelling density. Further, increases in PA associated with changes in the built environment are likely to be sustained, whereas the effectiveness of an efficacious behavioral intervention is often compromised by limitations in program reach, adoption, implementation, and maintenance (Ogilvie et al. 2007). In reality, PA interventions will reach only segments of a population, for a limited time, with limited sustained behavior change. But how feasible is it to modify the built environment to increase PA by the magnitude indicated by our analyses? To consider dwelling density across the cities in our study, a 1-SD change in gross dwelling density (inclusive of all amenities such as streets, schools, and green spaces) would mean an increase from 19 to 59 gross dwellings per hectare, a figure consistent with guidelines for developing mixed-use, transit- oriented neighborhoods (Churchman 1999). Urban change of this magnitude is more likely in greenfield (land not previously built on) and brownfield (previously industrial land) sites in the short term, with retrofitting existing neighborhoods a longer-term agenda. There will be unique circumstances such as Christchurch, New Zealand, where large-scale urban reconstruction after major earthquakes in 2010 and 2011 will provide an opportunity to design medium-density, mixed-use neighborhoods.

The health benefits of a more compact urban form are likely to extend beyond increasing population levels of PA and downstream improvement in chronic disease prevalence. As Woodcock et al. (2009) reported, an increase in active travel, if accompanied by a decline in carbon-emitting motorized travel, will also benefit health through reduced air pollution. To encourage a higher uptake of active travel, Woodcock et al. (2009) called for “policies to increase the acceptability, appeal and safety of active urban travel.” However our study suggests that to achieve the potential health and environmental co-benefits of increasing all forms of PA, the morphology of urban neighborhoods, as well as the quality of streetscapes, needs attention.

## Conclusions

A consistent pattern of positive associations was observed between built environment characteristics (street connectivity, destination accessibility, and dwelling density) and transport and leisure-related PA outcomes. Further, the study found these associations were largely unchanged after controlling for participants’ preference for living in a more or less walkable neighborhood. The study adds strength to the growing international evidence that there is a substantial opportunity to increase population level PA, for transport and leisure, through structural changes to our built environments.

## Supplemental Material

(115 KB) PDF

## References

[r1] Badland H, Opit S, Witten K, Kearns R, Mavoa S. (2010). Can virtual streetscape audits reliably replace physical streetscape audits?. J Urban Health.

[r2] Badland H Schofield G Witten K Schluter P Mavoa S Kearns R 2009 Understanding the Relationship between Activity and Neighbourhoods (URBAN) Study: research design and methodology. BMC Public Health 9 224; doi:10.1186/1471-2458-9-224 [Online 10 July 2009] 19589175 PMC2716337

[r3] Brownson RC, Hoehner CM, Day K, Forsyth A, Sallis JF (2009). Measuring the built environment for physical activity: state of the science.. Am J Prev Med.

[r4] Chaix B, Merlo J, Evans D, Leal C, Havard S. (2009). Neighbourhoods in eco-epidemiologic research: delimiting personal exposure areas. A response to Riva, Gauvin, Apparicio, and Brodeur.. Soc Sci Med.

[r5] Churchman A. (1999). Disentangling the concept of density.. J Plan Lit.

[r6] Conn V, Hafdahl A, Mehr D. (2011). Interventions to increase physical activity among healthy adults: meta-analysis of outcomes.. Am J Public Health.

[r7] Craig C, Marshall A, Sjostrom M, Bauman A, Booth M, Ainsworth B (2003). International physical activity questionnaire: 12-country reliability and validity.. Med Sci Sport Exerc.

[r8] De Bourdeaudhuij J, Sallis JF, Saelens BE (2003). Environmental correlates of physical activity in a sample of Belgian adults.. Am J Health Promot.

[r9] Ewing R. (2009). Travel and the built environment: a meta-analysis.. J Am Plan Assoc.

[r10] Frank LD, Andresen MA, Schmid TL (2004). Obesity relationships with community design, physical activity, and time spent in cars.. Am J Prev Med.

[r11] Frank LD, Saelens BE, Powell KE, Chapman JE (2007). Stepping towards causation: do built environments or neighborhood and travel preferences explain physical activity, driving, and obesity?. Soc Sci Med.

[r12] Frank LD, Sallis JF, Saelens BE, Leary L, Cain K, Conway TL (2010). The development of the walkability index: application to the Neighborhood Quality of Life Study.. Br J Sport Med.

[r13] Frank LD, Schmid TL, Sallis JF, Chapman J, Saelens BE (2005). Linking objectively measured physical activity with objectively measured urban form: findings from SMARTRAQ.. Am J Prev Med.

[r14] Giles-Corti B, Timperio A, Bull F, Pikora T. (2005). Understanding physical activity environmental correlates: increased specificity for ecological models.. Exerc Sport Sci Rev.

[r15] IPEN (International Physical Activity and the Environment Network) (2001). IPEN Homepage.. http://www.ipenproject.org/.

[r16] Leslie E, Coffee N, Frank L, Owen N, Bauman A, Hugo G. (2007). Walkability of local communities: using geographic information systems to objectively assess relevant environmental attributes.. Health Place.

[r17] Levine J, Frank L. (2007). Transportation and land-use preferences and residents’ neighborhood choices: the sufficiency of compact development in the Atlanta region.. Transportation.

[r18] Levine J, Inam A, Torng G-W. (2005). A choice-based rationale for land use and transportation alternatives: evidence from Boston and Atlanta.. J Plan Educ Res.

[r19] Ministry of Transport (2009). How New Zealanders Travel: Trends in New Zealand Household Travel 1989–2008.

[r20] Ogilvie D Foster CE Rothnie H Cavill N Hamilton V Fitzsimons CF 2007 Interventions to promote walking: systematic review. BMJ 334 1204; doi:10.1136/bmj.39198.722720.BE [Online 7 June 2007] 17540909 PMC1889976

[r21] Owen N, Cerin E, Leslie E, duToit L, Coffee N, Frank LD (2007). Neighborhood walkability and the walking behavior of Australian adults.. Am J Prev Med.

[r22] Owen N, Humpel N, Leslie E, Bauman A, Sallis J. (2004). Understanding environmental influences on walking: review and research agenda.. Am J Prev Med.

[r23] Pikora TJ, Bull FC, Jamrozik J, Knuiman M, Giles-Corti B, Donovan RJ (2002). Developing a reliable audit instrument to measure the physical environment for physical activity.. Am J Prev Med.

[r24] Saelens BE, Glanz K (2009). Work group I: measures of the food and physical activity environment.. Am J Prev Med.

[r25] Saelens B, Handy S. (2008). Built environment correlates of walking: a review.. Med Sci Sport Exerc.

[r26] Sallis JF (2009). Measuring physical activity environments: a brief history.. Am J Prev Med.

[r27] Sallis JF, Bowles HR, Bauman A, Ainsworth BE, Bull FC, Craig CL (2009a). Neighborhood environments and physical activity among adults in 11 countries.. Am J Prev Med.

[r28] Sallis JF, Saelens BE, Frank LD, Conway TL, Slymen DJ, Cain KL (2009b). Neighborhood built environment and income: examining multiple health outcomes.. Soc Sci Med.

[r29] Salmond C, Crampton P, Atkinson J (2007). NZDep2006 Index of Deprivation..

[r30] Sport and Recreation New Zealand (2008). Sport, Recreation and Physical Activity Participation among New Zealand Adults: Key Results of the 2007/08 Active New Zealand Survey..

[r31] Story M, Giles-Corti B, Yaroch AL, Cummins S, Frank LD, Huang TT (2009). Work group IV: future directions for measures of food and physical activity environments.. Am J Prev Med.

[r32] Transportation Research Board and Institute of Medicine of the National Academies (2005). Does the Built Environment Influence Physical Activity? Examining the Evidence. TRB Special Report 282.

[r33] Van Dyck D, Cardon G, Deforche B, Sallis JF, Owen N, De Bourdeaudhuij I (2010). Neighborhood SES and walkability are related to physical activity behavior in Belgian adults.. Prev Med.

[r34] Wendel-Vos W, Droomers M, Kremers S, Brug J, van Lenthe F. (2007). Potential environmental determinants of physical activity in adults: a systematic review.. Obes Rev.

[r35] Witten K, Pearce J, Day P. (2011). Neighbourhood Destination Accessibility Index: a GIS tool for measuring infrastructure support for neighbourhood physical activity.. Environ Plan A.

[r36] Woodcock J, Edwards P, Tonne C, Armstrong B, Ashiru O, Banister D (2009). Public health benefits of strategies to reduce greenhouse-gas emissions: urban land transport.. Lancet.

