# Supplementary material for: Neighborhood Built Environment and Transport and Leisure Physical Activity: Findings Using Objective Exposure and Outcome Measures in New Zealand
Source: Environ Health Perspect. 2012 Mar 28;120(7):971–7. doi: 10.1289/ehp.1104584 (PMC3404665; doi:10.1289/ehp.1104584)
Supplement: (115 KB) PDF [file ehp.1104584.s001.pdf]

Supplemental Material

Neighbourhood Built Environment and Transport and Leisure Physical Activity: New Zealand Findings Using Objective Exposure and Outcome Measures

Karen Witten, Tony Blakely, Nasser Bagheri, Hannah Badland, Vivienne Ivory

Jamie Pearce, Suzanne Mavoa, Erica Hinckson, Grant Schofield

Table of contents

Supplemental Material, Table 1: Odds ratios (95% CI) for any self-reported transport, walking (all purposes), or leisure related physical activity (versus no self-reported physical activity),for a one standard deviation change in each neighbourhood exposure 2

Supplemental Material, Table 2: Predicted relative change (95% CI) in self-reported (among those with some self-reported activity) and accelerometer-measured physical activity, for a one standard deviation change in neighbourhood exposures 3

	Odds ratios for any self-reported PA versus none[a b] (95% CI)		
Neighbourhood exposure	Transport	Leisure	Walking
1. Adj. demographics	n=2017	n=2018	n=2015
Streetscape	1.12 (0.98-1.28)	1.03 (0.89-1.18)	1.08 (0.89-1.29)
NDAI	1.37 (1.14-1.65)	1.10 (0.90-1.35)	1.40 (1.11-1.78)
Street connectivity	1.38 (1.16-1.63)	1.04 (0.85-1.26)	1.23 (0.98-1.54)
Dwelling Dens	1.32 (1.09-1.60)	1.05 (0.85-1.30)	1.22 (0.97-1.55)
Mixed land use	1.10 (0.87-1.39)	1.19 (0.96-1.49)	1.09 (0.83-1.44)
2.Plus adj. individual-level socioeconomic factors	n=1804	n=1806	n=1803
Streetscape	1.12 (0.97-1.29)	1.02 (0.88-1.17)	1.09 (0.90-1.31)
NDAI	1.29 (1.07-1.56)	1.15 (0.95-1.37)	1.37 (1.08-1.72)
Street connectivity	1.30 (1.11-1.54)	1.08 (0.90-1.29)	1.23 (0.99-1.53)
Dwelling Dens	1.25 (1.04-1.50)	1.09 (0.90-1.32)	1.22 (0.98-1.53)
Mixed land use	1.02 (0.81-1.27)	1.20 (0.98-1.47)	1.00 (0.77-1.31)
3. Plus adj. for neighbourhood deprivation	n=1804	n=1806	n=1803
Streetscape	1.13 (0.98-1.30)	1.03 (0.89-1.18)	1.10 (0.93-1.31)
NDAI	1.39 (1.15-1.69)	1.28 (1.06-1.54)	1.45 (1.17-1.79)
Street connectivity	1.41 (1.18-1.68)	1.21 (1.00-1.47)	1.30 (1.05-1.62)
Dwelling Dens	1.36 (1.10-1.67)	1.22 (0.99-1.50)	1.25 (0.99-1.57)
Mixed land use	1.03 (0.82-1.29)	1.23 (1.01-1.50)	1.02 (0.80-1.28)

Supplemental Material, Table 1: Odds ratios (95% CI) for any self-reported transport, walking (all purposes), or leisure related physical activity (versus no self-reported physical activity), for a one standard deviation change in each neighbourhood exposure

[a] From models of ln[IPAQ-minutes] on neighbourhood exposures (transformed to have standard deviation of 1.0) and covariates, whereby the exponential of the coefficient of the neighbourhood exposure is the ratio change in any minutes of self-reported physical activity.

[b]Estimates were generated using multi-level logistic regression models with covariates age (categories of Age bands: 15-29yrs, 30-44yrs (reference group), 45-54yrs, 55-65yrs), ethnicity (Māori, non-Māori (reference group)), sex (male (reference group), female), qualifications (No Qualification, School, Post School, Tertiary (reference group)), marital status (Never married, Married (reference group), Previous married) household income (<$ 40,000, $ 40,001-60,000, $ 60,001-80,000, $ 80,001-100,000, >$ 100,000 (reference group)) , employment (Fulltime work (reference group), Part-time/not working), car access (Un restricted (reference group), Restricted, No car access) , neighbourhood deprivation (New Zealand Deprivation Index 2006 Quintiles 1 (less deprived) (reference group) to 5 (most deprived) and neighbourhood preferences (Strongly prefer walkable, Moderately prefer walkable, Neutral (reference group), Moderately prefer less walkable, Strongly prefer less walkable).

Neighbourhood exposure	Relative change in self-reported minutes of PA (95% CI)[a b] Transport	Leisure	Walking	Relative change in accelerometer count Weekday	Weekend
1.Adj. demographics	n=1404	n=1498	n=1793	n=1823	n=1704
Streetscape	1.04 (0.97-1.12)	1.07 (1.00-1.14)	1.06 (0.98-1.15)	1.03 (1.00-1.06)	1.02 (0.98-1.06)
NDAI	1.16 (1.06-1.27)	1.06 (0.97-1.15)	1.19 (1.07-1.32)	1.04 (1.00-1.08)	1.01 (0.96-1.06)
Street connectivity	1.13 (1.03-1.24)	1.06 (0.97-1.15)	1.12 (1.00-1.24)	1.04 (1.00-1.08)	1.03 (0.98-1.08)
Dwelling Dens	1.15 (1.04-1.27)	1.03 (0.94-1.13)	1.14 (1.02-1.28)	1.04 (1.00-1.08)	1.02 (0.97-1.08)
Mixed land use	1.08 (0.97-1.22)	1.09 (0.98-1.20)	1.08 (0.95-1.24)	1.04 (0.99-1.09)	1.04 (0.98-1.10)
2.Plus adj. individual-level socioeconomic factors	n=1253	n=1339	n=1600	n=1641	n=1534
Streetscape	1.05 (0.98-1.13)	1.06 (0.99-1.13)	1.07 (0.99-1.16)	1.03 (1.00-1.07)	1.01 (0.97-1.05)
NDAI	1.13 (1.03-1.24)	1.07 (0.98-1.17)	1.15 (1.03-1.28)	1.04 (1.00-1.08)	1.01 (0.97-1.06)
Street connectivity	1.12 (1.03-1.23)	1.08 (1.00-1.18)	1.09 (0.98-1.22)	1.04 (1.00-1.08)	1.03 (0.99-1.07)
Dwelling Dens	1.14 (1.04-1.25)	1.05 (0.95-1.15)	1.12 (0.99-1.26)	1.04 (1.00-1.08)	1.03 (0.98-1.07)
Mixed land use	1.07 (0.96-1.19)	1.08 (0.98-1.19)	1.07 (0.94-1.22)	1.03 (0.99-1.08)	1.03 (0.98-1.08)
3. Plus adj. for neighbourhood deprivation	n=1253	n=1339	n=1600	n=1641	n=1534
Streetscape	1.05 (0.98-1.13)	1.06 (0.99-1.13)	1.07 (0.99-1.16)	1.03 (1.00-1.07)	1.01 (0.98-1.05)
NDAI	1.09 (0.99-1.21)	1.11 (1.01-1.22)	1.13 (1.00-1.28)	1.07 (1.03-1.11)	1.05 (1.00-1.10)
Street connectivity	1.08 (0.98-1.19)	1.13 (1.03-1.25)	1.06 (0.93-1.21)	1.07 (1.02-1.11)	1.07 (1.02-1.11)
Dwelling Dens	1.10 (0.98-1.23)	1.09 (0.98-1.21)	1.09 (0.95-1.25)	1.07 (1.03-1.12)	1.07 (1.02-1.12)
Mixed land use	1.05 (0.94-1.17)	1.10 (0.99-1.22)	1.08 (0.94-1.24)	1.04 (0.99-1.09)	1.04 (0.99-1.09)

Supplemental Material, Table 2: Predicted relative change (95% CI) in self-reported (among those with some self-reported activity) and accelerometer-measured physical activity, for a one standard deviation change in neighbourhood exposures

IPAQ=International Physical Activity Questionnaire, PA=Physical Activity NDAI= Neighbourhood Destination Accessibility Index

[a] From models of ln[IPAQ-minutes] or ln[accelerometer count] on neighbourhood exposures (transformed to have standard deviation of 1.0) and covariates, whereby the exponential of the coefficient of the neighbourhood exposure is the ratio change of increase in accelerometer count.

[b] Estimates were generated using multi-level linear regression models with covariates age (categories of Age bands: 15-29yrs, 30-44yrs (reference group), 45-54yrs, 55-65yrs), ethnicity (Māori, non-Māori (reference group)), sex (male (reference group), female), qualifications (No Qualification, School, Post School, Tertiary (reference group)), marital status (Never married, Married (reference group), Previous married), household income (<$ 40,000, $ 40,001-60,000, $ 60,001-80,000, $ 80,001-100,000, >$ 100,000 (reference group)) , employment (Fulltime work (reference group), Part-time/not working), car access (Un restricted (reference group), Restricted, No car access) , neighbourhood deprivation (New Zealand Deprivation Index 2006 Quintiles 1 (less deprived) (reference group) to 5 (most deprived)) and neighbourhood preferences (Strongly prefer walkable, Moderately prefer walkable, Neutral (reference group), Moderately prefer less walkable, Strongly prefer less walkable).
